# Radiofrequency thermocoagulation as a treatment for hemifacial spasm: long-term follow-up and management of recurrences

**DOI:** 10.1007/s00701-024-06149-0

**Published:** 2024-06-15

**Authors:** Paula Palomäki, Tiina Sairanen, Mika Niemelä, Johan Marjamaa

**Affiliations:** 1 Hemifacial Spasm Research Group, Helsinki University Hospital, HUS Neurocenter, Helsinki, Uusimaa Finland; 2 Department of Neurology, Helsinki University Hospital, Helsinki, Uusimaa Finland; 3 Department of Neurosurgery, Helsinki University Hospital, Helsinki, Uusimaa Finland

**Keywords:** Radiofrequency thermocoagulation, Hemifacial spasm, Paresis, Recurrence

## Abstract

**Background:**

Radiofrequency thermocoagulation (RFT) is a treatment used to relieve symptoms of cranial nerve disorders. The current study is the first to describe the results of hemifacial spasm (HFS) patients with a history of repeated RFT in the second-largest consecutive single-center patient series with long-term follow-up.

**Method:**

This retrospective study was conducted in the largest hospital district in Finland (Helsinki and Uusimaa). Consecutive HFS patients who had an RFT to treat HFS in the Hospital District of Helsinki and Uusimaa between 2009-2020 were included.

**Results:**

Eighteen patients with 53 RFTs were identified from the medical records. 11 (61 %) patients had repeated RFTs, and the mean number of RFTs per patient was 3.33 (3.29 SD). The mean follow-up was 5.54 years (7.5 SD). 12 (67 %) patients had had microvascular decompression (MVD) before RFT.

Patients were satisfied with the results after 87 % of RFTs. Relief of the twitching of the face lasted 11.27 months (11.94 SD). All patients had postoperatively transient facial paresis. Postoperative paresis lasted a mean of 6.47 months (6.80 SD). The depth of paresis was postoperatively typically moderate (36.54 %, House Brackmann III). 23.08 % had mild paresis (House-Brackmann II), 23.08 % had moderately severe dysfunction (House-Brackmann IV), 9.62 % had severe dysfunction, and 7.69 % had total paralysis of the facial muscles (House-Brackmann VI). Duration of relief in the face twitching (p 0.002) and temperature at the final coagulation point (p 0.004) were statistically significant predictors of satisfaction with the RFT results.

**Conclusions:**

RFT can be used to treat recurrences of HFS repeatedly. It provides symptom relief for around 11 months, lasting four times longer than with botulinum toxin injections. Patients are satisfied, although an RFT produces transient, sometimes even severe, facial paresis.

## Introduction

Hemifacial spasm (HFS) is a movement disorder of muscles innervated by the facial nerve, which is shown as progressive paroxysmal synchronous involuntary tonic and clonic contractions of facial muscles[12]. The mean annual age-standardized incidence of HFS is 1.53, and the mean age-standardized yearly prevalence is 10.62 per 100,000 people [13]. Incidence and prevalence of HFS grow progressively with age, peaking at the age group of eighty years and older [13]. HFS is more frequent among women and is typically left-sided [1, 13, 16].

Well-known treatment options for HFS are limited. Oral medications work poorly [4]. Botulinum toxin injections are effective and safe, but treatment results last approximately only three months [2]. Microvascular decompression (MVD) is known for potentially long-lasting results, but not all patients benefit from it [5, 11]. Radiofrequency thermocoagulation (RFT) has been rediscovered as an operative treatment option with long-lasting results for patients who did not benefit from MVD.

RFT is better known as a treatment option for trigeminal neuralgia and glossopharyngeal facial pain syndrome [8, 10, 15]. The first three small studies (7-27 patients) covering the original RFT technique for HFS were published between 1978 and 1987 [6, 9, 14]. After a long publication break, two more recent articles from 2021 (53 patients) and 2022 (83 patients) focused on a modified approach involving CT guidance during the operation [3, 7].

This article describes the long-term results of RFT in treating HFS.

## Methods and materials

### Patient population

Approval for this study was obtained in November 2019. Informed consent from the patients was waived due to the retrospective nature of this study. All consecutive patients with ICD-10 diagnoses of G51.31, G51.32, and G51.33 and who had a RFT of the facial nerve done to treat HFS between 2009 and 2020 were identified from the electronic medical records of the Department of Neurosurgery in HUS (Hospital District of Helsinki and Uusimaa). Clinical background information and treatment path from early symptoms and diagnosis up to 2020 were collected for each HFS patient. All HFS diagnoses were confirmed by a neurologist, otolaryngologist, or neurosurgeon with a particular interest in movement disorders. Microvascular decompression was considered a first-line surgical treatment option for all hemifacial spasm patients, and all patients were informed about it if their status allowed for general anesthesia and craniotomy. After microvascular decompression, all patients are evaluated postoperatively in an outpatient clinic for repeated microvascular decompression if the primary microvascular decompression did not give the desired results and whether RFT was considered a secondary surgical treatment option.

## Radiofrequency thermocoagulation treatment protocol

Only a few neurosurgeons in HUS are responsible for RFTs of HFS patients. RFT planning starts with a preoperative CT (computed tomography scan) to confirm the anatomy of the operation site. No routine intraoperative imaging was used. The patient is positioned in a lateral decubitus position over a pillow. A grounding electrode and other leads are placed. Light sedation is typically given intravenously before starting the operation. The puncture site of the skin is located 4-5 cm caudal from the mastoid along the anterior side of m. sternocleidomastoideus and the skin is injected subcutaneously with a local anesthetic.

A small incision with a knife is made to the puncture site, and the stylet is advanced through the puncture site towards stylomastoid foramen at first with the guidance of bony landmarks. The aim is to get the tip of the stylet as close as possible to the facial nerve in the stylomastoid foreman without causing mechanical damage to the nerve. The mandrin of the stylet is replaced by a radio frequency stimulation probe, and low-current (2 Hz, current 8.0 mA, 0.1-0.3 V) is applied to stimulate motor response to muscles innervated by the facial nerve. Rhythmic twitches of the ipsilateral facial muscles confirm the correct position of the stylet’s tip in proximity to the peripheral facial nerve. Suppose electric stimulation does not elicit a motor response. In that case, the radio frequency stimulation probe is replaced by the mandrin, and the position of the stylet is adjusted until electric stimulation elicits facial muscle twitches. Also, higher stimulation voltage up to and above 1.0 V may be used, but the motor response does not necessarily indicate that the stylet’s tip is close enough to the facial nerve.

Then, a radiofrequency probe is inserted into the stylet, and radiofrequency waves are applied. Radiofrequency waves are typically 20 seconds long. The starting temperature is 70° C and is increased in 5-10 °C increments up to 80 °C. During and between RFT, the patient is asked to engage facial muscles. The procedure is concluded once the patient struggles with shutting eyes or bulging cheeks. Slight weakness in facial muscles is desirable. Patients are kept for at least six hours for postoperative monitoring before leaving the hospital.

## Data collection

STROBE checklist was used to ensure this paper meets EQUATOR Network Reporting Guidelines. Patient demographics were collected regarding sex, side of the spasm, number of RFTs, length of follow-up, age of diagnosis, and HFS treatment allocations. Preoperative, intraoperative, and postoperative data were collected from each RFT: Preoperatively ASA class (The American Society of Anesthesiologists class). Intraoperative data included parameters of surgery technique and duration of surgery. Postoperative data contained patient-reported treatment satisfaction, duration of hospitalization, follow-up visit number, degree and duration of postoperative paresis, duration of symptom relief, and complications.

The duration of symptom relief was observed, and patients were asked about it postoperatively and during follow-up visits. Symptom relief was also considered to end when the patient contacted the Department of Neurosurgery asking for an evaluation for reoperation or botulinum toxin injections were restarted. Postoperative facial paresis was not considered a complication of the surgery. Patient-reported satisfaction was registered after every EC.

## Statistical analysis

The statistical analysis was performed using IBM SPSS Statistics v28.0 and Microsoft Excel v2018 software. Counts (N) and percentages (%) were used to describe the categorical variables. The mean with standard deviation (SD) was calculated to define continuous variables. In inferential statistics, p<0.05 was used as a threshold for statistically significant differences between groups. Two categorical variables with two groups were analyzed using Fisher’s exact test. Pearson chi-squared was used if the categorical variables had more than two groups. Before examining the distribution of continuous variables between two groups, the normality of the continuous variable was tested with Shapiro-Wilk, and the unity of variances was tested with Levine’s test. Then, according to the results, two samples of t-tests and Cohen’s D or Mann Whitney U with Partial Eta squared were applied. Pearson correlation was used to analyze the correlation of two continuous variables. Kaplan-Meier curves were used to analyze time-to-event relationships in the data.

The patient was considered overall satisfied with the results of RFTs if, on average, they were pleased with the results during the individual control visits. Parameters for inferential analysis of patient satisfaction and the duration of paresis were selected based on previous literature about the surgical technique [6, 7] and the expert opinion of co-author neurosurgeon Johan Marjamaa.

## Results

Eighteen patients had 53 completed RFTs of the facial nerve during the eleven-year study period. Additionally, seven operations were interrupted since the appropriate stimulation point of the facial nerve was not found (3 cases) or RFT did not cause paresis (4 cases). Interrupted operations were excluded from the analysis. Table 1 presents the clinical data of the HFS patients treated with RFT. 55.56 % were males. The side of the spasm was in 52 (94.44 %) cases left-sided. 11 (61 %) patients had more than one RFT for HFS, and during the mean follow-up time of 5.54 years (SD 7.5), patients underwent a mean of 3.33 (SD 3.29) RFTs. All patients had tested botulinum toxin injections in their treatment history. 55.56 % had also previously undergone microvascular decompression. For eight patients (44.44 %) in this cohort, RFT was chosen as the primary surgical treatment option for HFS. In some cases, the patient's status did not allow general anesthesia. In other ones, the patient made an informed decision to go for RFT as a minor operation, not requiring general anesthesia and easier recovery, despite being informed that microvascular decompression is far superior in the long-term results with a very low risk of permanent complications. The mean delay from diagnosis to the first RFT was 5.51 years (SD 3.55), and the first RFT was done at the mean age of 55.85 years (SD 15.35). In three-month control visits, 93 % of patients were, on average, satisfied with all past RFTs.

**Table 1:** Clinical data of 18 patients who underwent radiofrequency thermocoagulation treatment

Case	Sex	Side of Spasm	No of RFTs	Follow-up (years)	Oral medication*	BTX^a^	MVD^a^	NVC	Diagnosis age	Age at first RFT	Satisfaction^b^
1	f	left	4	3.04	no	yes	no	-	75	82	yes
2	f	left	2	24.05	yes	yes	yes	no	29	29	-
3	m	left	1	0.55	no	yes	yes	yes	67	72	yes
4	f	left	1	0.76	no	yes	no	-	56	69	-
5	f	left	5	5.85	no	yes	yes	yes	34	38	yes
6	m	left	3	12.84	no	yes	no	-	41	48	yes
7	m	left	10	7.28	yes	yes	yes	no	30	32	yes
8	m	left	1	0.75	yes	yes	yes	yes	49	53	-
9	f	left	5	6.24	no	yes	yes	-	41	47	yes
10	f	left	1	0.47	yes	yes	yes	yes	46	49	yes
11	m	left	2	2.10	no	yes	yes	-	62	67	yes
12	m	left	1	0.13	yes	yes	yes	-	52	59	no
13	m	left	1	0.23	yes	yes	no	-	65	67	yes
14	f	left	1	0.00	no	yes	yes	no	54	59	-
15	m	left	11	18.37	no	yes	yes	-	48	59	yes
16	m	left	1	0.16	no	yes	no	-	62	70	yes
17	f	right	1	0.25	yes	yes	no	-	59	62	yes
18	m	left	2	16.58	no	yes	yes	yes	53	53	yes

^a^History of X treatment for Hemifacial spasm before first RFT

^b^Overall satisfaction with radiofrequency thermocoagulation treatment results

Abbreviations of the table: *No* number, *RFT* radiofrequency thermocoagulation, *BTX* botulinum toxin, *MVD* microvascular decompression, *f* female, *m* male, - data missing, *NVC* Neurovascular compression detected in MRI

**Fig. 1:**
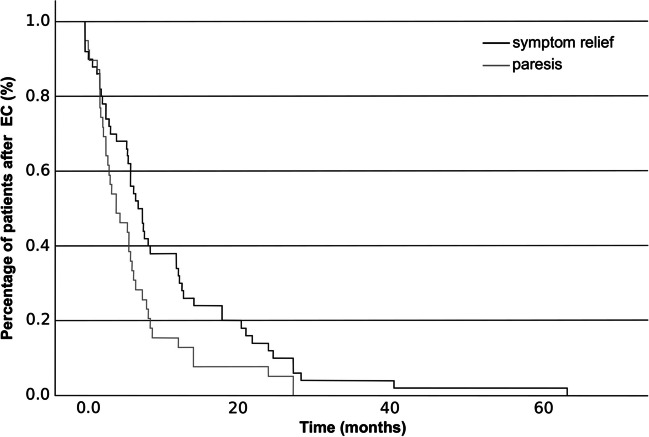
Kaplan-Meier curves represent the percentage of hemifacial spasm patients gaining relief from the twitching of the face and having paresis after radiofrequency thermocoagulation treatment of the facial nerve. This figure was created by using Inkscape

Table 2 shows pre-, intra, and postoperative data collected from the 53 RFTs. When asked after each RFT in control visit, 87 % of patients were satisfied with the results. Duration of relief in the twitching of the face lasted a mean of 11.27 months (SD 11.94), while postoperative paresis lasted a mean of 6.47 (SD 6.80) months, leaving 4.84 (SD 10.22) months free of twitching and paresis. All patients had postoperatively transient facial paresis. The depth of paresis was typically (36.54 %) moderate (House-Brackmann III). 23.08 % had mild paresis (House-Brackmann II), 23.08 % had moderately severe dysfunction (House-Brackmann IV), 9.62 % had severe dysfunction, and 7.69 % had total paralysis of the facial muscles (House-Brackmann VI). After 12 (24 %) RFTs, patients experienced complications or other perceived side effects postoperatively, including dry eve, facial pain, ectropium, or altered sense of touch on the face. Figure 1 presents the duration of paresis and duration of relief in the twitching of the face after RFT.

**Table 2:** 53 radiofrequency thermocoagulation treatments between 1995-2020

Variable	N (%)
Satisfied with the results in the control visit*	26 (86.67)
House-Brackmann (pop)*^1^	
- I	0 (0.00)
- II	12 (23.08)
- III	19 (36.54)
- IV	12 (23.08)
- V	5 (9.62)
- VI	4 (7.69)
Complications or other perceived side effects postoperatively*^2^	
- dry eye	4 (8.16)
- facial pain	4 (8.16)
- ectropium	3 (6.12)
- altered sense of touch on the face	1 (2.04)
**Variable**	**Mean (SD)**
Duration of relief in the twitching of the face (months)*^3^	11.27 (11.94)
Duration of paresis (months)*^4^	6.47 (6.80)
Age at the time of operation (years)	55.86 (15.35)
Delay of operation from diagnosis (years)	9.91 (7.01)
Duration of the surgery (minutes)*^5^	42 (31)
Time from surgery to follow-up visit (months)*^6^	3.49 (2.07)
Voltage of stimulation that caused contractions in the final coagulation point (V)*^7^	0.36 (0.17)
The temperature at the final coagulation point (°C)*^8^	70.98 (6.22)
Coagulation time at final point (s)*^9^	41.65 (0.24)

^*^ 23 (43.40 %) datapoints missing

^*^^1^ 1 (1.89 %) datapoints missing

^*^^2^ 4 (7.55 %) datapoints missing

^*^^3^ 4 (7.55 %) datapoints missing

^*^^4^ 14 (26.42 %) datapoints missing

^*^^5^ 5 (9.43 %) datapoints missing

^*^^6^ 9 (16.98 %) datapoints missing

^*^^7^ 14 (26.42 %) datapoints missing

^*^^8^ 8 (15.09 %) datapoints missing

^*^^9^ 7 (13.21 %) datapoints missing

When analyzing pre-, intra-, and postoperative factors associated with patient-reported satisfaction, the duration of relief in the twitching of the face (p 0.002) correlated positively, and temperature at the final coagulation point (p 0.004) correlated negatively. Other parameters (Table 3**)** were not associated with patient satisfaction. Furthermore, the duration of postoperative paresis correlated positively with the duration of relief in the twitching of the face (p 0.002). Other parameters (Table 4**)** were not associated with the paresis duration.

**Table 3:** Demographic, perioperative, and follow-up parameters correlation with patient satisfaction after radiofrequency thermocoagulation treatment

Variable	P-value	Group means (difference)
Sex	.611	-
Side of Spasm	1.000	-
Age at the time of radiofrequency thermocoagulation treatment	.088	-
Coagulation time at final point (s)	.102	-
Temperature at final coagulation point (°C)	.004	69.71, 77.50 (-7.79)
Coagulation time at final point (s)	.102	-
Voltage of stimulation that caused contractions in the final point (v)	0.055	-
Duration of paresis	.104	-
Duration of relief in the twitching of the face (months)	.002	15.32, 0.41 (14.91)
Depth of postoperative paresis	.093	-
Postoperative complications (categorical)	1.000	-
Postoperative complications (numerical)	.826	-

**Table 4:** Demographic, perioperative, and follow-up parameters correlation with the duration of postoperative paresis

Variable	P-value	Pearson correlation
Age at the time of radiofrequency thermocoagulation treatment	.848	-
Voltage of stimulation that caused contractions in the final coagulation point (V)	.752	-
Temperature at final coagulation point (°C)	.444	-
Coagulation time at final point (s)	.118	-
Duration of relief in the twitching of the face (months)	.002	0.490

## Discussion

This study is the first to report long-term results of consecutive RFT cases with a mean follow-up of 5.54 years per patient. Previously, the most extended study period was three years, reported in 2022 by Du et al. [3]. In 1981, Hori et al. reported follow-ups of their patients for up to 1.5 years with 28 operations, including one patient with repeated RFT [6]. Furthermore, with 53 RFTs, this is the 2^nd^ largest study reporting RFT results for HFS. Du et al. have previously published results of 82 patients in 2022 [7], and Huang et al. of 53 patients in 2021.^14^ The present study is also unique in the sense that it is the first to show that RFT can be done multiple times for the same patient. One patient had up to eleven RFTs during the study period.

On average, 93 % of the patients were satisfied with the results of follow-up visits. This aligns with a previous study in which only two patients (3.8 %) reported dissatisfaction with the treatment results [7]. Furthermore, 11 (61 %) patients in the current study underwent RFT again. A similar proportion of patients, namely 10 (56 %), had previously had MVD. Complications did not correlate with patient satisfaction. Overall, these findings may imply that the history of other treatment options or previous RFTs does not seem to be an obstacle for reoperation, and the adverse events (dry eye, facial pain, ectropium, altered sense of touch) are tolerable compared to the benefits of RFT.

The duration of relief in the twitching of the face correlated positively with patient satisfaction after RFT. Moreover, the duration of postoperative paresis correlated positively with the duration of relief in the twitching of the face. This might imply that longer-lasting paresis might be connected to more long-lasting results and higher patient satisfaction. Before going for RFT, all patients are informed about postoperative paresis as an inevitable side effect that might alter the patients' expectations compared to microvascular decompression. Furthermore, lower coagulation temperatures might also be desirable for a better patient-related experience. In the current study, patients with a mean temperature of 70°C at the final coagulation point were more pleased with the results than the group with temperatures eight Celsius higher.

In the current series, the duration of relief in the twitching of the face lasted 11 months, with a mean follow-up of 5.54 years (SD 7.5) per patient. In line with this study, Huang et al. reported five patients (9.4 %) with recurrence of symptoms after the mean follow-up period of eleven months [7]. Salar et al. and Hori et al. did not have any recurrences within their study period of 10 months and 1.5 years [6, 14]. These differences suggest that the results of RFT are not typically everlasting, but the durations vary. The expected benefit time from RFT is at least four times longer than usually from botulinum toxin injections.

In this cohort, postoperative paresis lasted a mean of 6.47 months (6.80 SD), and the depth of paresis was postoperatively typically moderate in 36.5 % of patients (House Brackmann III). In the series of Huang et al., postoperative paresis resolved within one month, yet the depth of paresis was postoperatively moderate in a larger proportion, i.e., 62 % of patients (House Brackmann III) [7]. Compared with paresis for up to 10 months (Salar et al.) and duration of paresis of 2.5 months (Hori et al.), our results are in between these cohorts [6, 14]. Overall, RFT-treated patients have postoperative facial weakness, which typically resolves in one to ten months.

The anatomical landmarks and electric low-current stimulation of the facial nerve’s motor response have been stable in detecting the optimal coagulation point of the facial nerve from the 1970s. Tested imaging methods include preoperative submentovertex skull x-ray, intraoperative fluoroscopy, and intraoperative intermittent CT guidance [6, 7]. Patients had preoperative cranial CT in the current series, but no routine intraoperative imaging was used. The mean duration of the surgery was 42 minutes (31 SD) in our cohort; meanwhile, with intraoperative imaging, Hori et al. and Huang et al. reported operation times of around 30 minutes [6, 7]. This suggests that intraoperative imaging techniques save operating room time.

A limitation of the present study is that it is a retrospective study based on the data from patients' medical records, so some data points were inevitable to be missing. However, the retrospective approach allows for studying rare conditions like HFS with long follow-up times time-efficiently. It is also worth noticing that Finland provides comprehensive public general and specialized health care services for Finnish citizens paid by taxes, not private insurance, including HFS treatment. Thus, most HFS patients are treated in public hospitals, making patient follow-up accurate.

RFT is a well-documented option in treating HFS with almost four times longer-lasting results than botulinum toxin injections, and complications are well tolerated. Patients are satisfied, although an RFT produces transient, sometimes even severe, facial paresis. Furthermore, RFT seems suitable regardless of previous treatment history of HFS and can be repeated multiple times if necessary. We see an indication for RFT if the results of botulinum toxin injections are unsatisfactory and one of the following: The status of the patient does not allow craniotomy and general anesthesia, a patient has a recurrence after MVD or the patient does not want to go under MVD after being informed that MVD is superior in the long-term results with a low rate of permanent complications.

## Data Availability

The data supporting this study's findings are not openly available to protect the privacy of study participants. However, they are available from the corresponding author upon reasonable request. The data are located in controlled access data storage at the University of Helsinki.

## Abbreviations

HFS: Hemifacial spasm
MVD: Microvascular decompression
RFT: Radiofrequency thermocoagulation
HUS: Hospital District of Helsinki and Uusimaa
CT: Computed tomography scan

## Code availability

This declaration is not applicable.

## Declarations

### Standards of reporting

STROBE checklist was used to ensure this paper meets EQUATOR Network Reporting Guidelines.
